# Development of an anti‐BAG3 humanized antibody for treatment of pancreatic cancer

**DOI:** 10.1002/1878-0261.12492

**Published:** 2019-05-17

**Authors:** Anna Basile, Margot De Marco, Michelina Festa, Antonia Falco, Vittoria Iorio, Luana Guerriero, Daniela Eletto, Domenica Rea, Claudio Arra, Alessia Lamolinara, Patrizia Ballerini, Verena Damiani, Alessandra Rosati, Gianluca Sala, Maria Caterina Turco, Liberato Marzullo, Vincenzo De Laurenzi

**Affiliations:** ^1^ BIOUNIVERSA s.r.l. R&D Division University of Salerno Baronissi Italy; ^2^ Department of Medicine, Surgery and Dentistry University of Salerno Baronissi Italy; ^3^ Department of Pharmacy University of Salerno Fisciano Italy; ^4^ S.S.D. Sperimentazione Animale Istituto Nazionale Tumori “IRCCS” Fondazione G. Pascale Naples Italy; ^5^ Dipartimento di Scienze Mediche Orali e Biotecnologiche Centro Studi sull'Invecchiamento CeSI‐MeT University ‘G. d'Annunzio’ di Chieti‐Pescara Italy; ^6^ Department of Neuroscience, Imaging and Clinical Sciences and Center for Research on Aging and Translational Medicine (CeSI‐MeT) ‘G. d'Annunzio’ University of Chieti Italy

**Keywords:** BAG3, humanized antibody, pancreatic cancer, pancreatic ductal adenocarcinoma, tumor therapy

## Abstract

We have previously shown that secreted BAG3 is a potential target for the treatment of pancreatic ductal adenocarcinoma and that pancreatic tumor growth and metastatic dissemination can be reduced by treatment with an anti‐BAG3 murine antibody. Here, we used complementarity‐determining region (CDR) grafting to generate a humanized version of the anti‐BAG3 antibody that may be further developed for possible clinical use. We show that the humanized anti‐BAG3 antibody, named BAG3‐H2L4, abrogates BAG3 binding to macrophages and subsequent release of IL‐6. Furthermore, it specifically localizes into tumor tissues and significantly inhibits the growth of Mia PaCa‐2 pancreatic cancer cell xenografts. We propose BAG3‐H2L4 antibody as a potential clinical candidate for BAG3‐targeted therapy in pancreatic cancer.

**Abbreviations**
BAG3: BCL2‐associated athanogene 3
CAF: cancer‐associated fibroblasts
CDR: complementarity‐determining region
H and L: heavy (H) and light (L) chain variants
Hsp: heat shock protein
IFITM‐2: Interferon‐Induced TransMembrane protein‐2
IL‐6: interleukin 6
MAP: multiple antigenic peptides
PD‐1: programmed cell death 1
PDAC: pancreatic ductal adenocarcinoma
TAM: tumor‐associated macrophages
α‐SMA: actin, alpha 2, smooth muscle, aorta

## Introduction

BAG3 protein, a cochaperone of the heat shock protein (Hsp) 70 (Takayama *et al*., 1999), interacts with several key molecules in cells, either via Hsp70 or, directly, via its WW domain, proline‐rich region (PXXP), and IPV (Ile‐Pro‐Val) motifs, thus regulating major cellular pathways such as apoptosis, autophagy, cytoskeleton organization, and cell motility (Ammirante *et al*., 2010, 2011; Boiani *et al*., 2013; Chiappetta *et al*., 2012; Falco *et al*., 2012; Festa *et al*., 2011; Kong *et al*., 2016; Rosati *et al*., 2011). BAG3 expression is induced in response to cell stress in a number of tissues (Du *et al*., 2015; Franceschelli *et al*. 2018; Pagliuca *et al*., 2003; Rosati *et al*. 2007; Rapino *et al*., 2014; Lee *et al*., 2002; Wang *et al*., 2012; and reviewed in De Marco *et al*., 2018), while it is constitutively expressed in cardiomyocytes where it is known to favor homeostasis during mechanical, proteotoxic, and other types of stress (De Marco *et al*., 2011, 2013, 2014; Hishiya *et al*., 2010; Homma *et al*., 2006; Judge *et al*., 2017; Mizushima and Sadoshima, 2017). Moreover, BAG3 defects impair myocyte survival or contractility resulting in heart myopathies (Belkaya *et al*., 2017; Fang *et al*., 2017).

A growing body of evidence suggests an important role for BAG3 in cancer development; indeed, BAG3 has been shown to be constitutively expressed in different types of cancer (Aust *et al*., 2013; Bartsch *et al*., 2016; Chiappetta *et al*., 2012; Chiappetta *et al*., 2014; De Marco *et al*., 2018; Esposito *et al*., 2017; Festa *et al*., 2011; Franco *et al*., 2012; Guerriero *et al*., 2014; Guerriero *et al*., 2017; Li *et al*., 2018; Nymoen *et al*., 2015; Rosati *et al*., 2012a,b; Shi *et al*., 2016; Shi *et al*., 2018; Yunoki *et al*., 2017; Yeo *et al*., 2015; Xiao *et al*., 2014) and to correlate with tumor aggressiveness in pancreatic adenocarcinomas (Rosati *et al*., 2012a,b), melanomas (Guerriero *et al*., 2014), primary serous ovarian cancers (Nymoen *et al*., 2015), and colorectal cancers (Li *et al*., 2018).

We also reported that BAG3 is released by pancreatic ductal adenocarcinoma (PDAC) cells (Rosati *et al*., 2015) and is detectable in serum samples from PDAC patients (Falco *et al*., 2013). PDAC‐released BAG3 binds via its receptor IFITM‐2 (Interferon‐Induced TransMembrane protein‐2) to macrophages activating them and inducing them to secrete factors that promote PDAC cell proliferation (Rosati *et al*., 2015). The block of this paracrine loop through an anti‐BAG3 antibody reduces tumor cell proliferation, tumor growth, and metastasis formation. Moreover, we have recently shown that anti‐BAG3 has an additive effect with anti‐PD1 in PDAC treatment in a syngeneic mouse model.

Here, we describe the development of a humanized BAG3‐blocking antibody and show its potential therapeutic effect in a murine model of PDAC.

## Methods

### Cell cultures

The murine macrophage cell line J774.A1 and pancreatic cancer cell lines (PANC‐1 and MIA PaCa‐2) were purchased from the American Type Culture Collection (ATCC, Manassas, VA, USA) and cultured in DMEM supplemented with 10% heat‐inactivated fetal bovine serum. MIA PaCa‐2 cells were also supplemented with 2.5% of horse serum (GIBCO, Life Technologies, Grand Island, NY, USA). Human peripheral blood mononuclear cells (PBMC) were isolated by Lymphocyte Separation Medium (Lonza, # 17‐829F, Basel, Switzerland) density gradient centrifugation. Monocytes (>98% CD14^+^) were isolated using the Monocyte Isolation Kit II (Miltenyi Biotec, Bergisch Gladbach, Germany) according to the manufacturer's protocol and cultured in RPMI‐1640 medium. Blood samples were collected in accordance with the project N.106546 approved from the local ethics committee. Cell cultures were maintained at 37 °C in a 5% CO_2_ atmosphere.

### BAG3 antibodies' KD assessment

Binding experiments were performed on Biacore 2000 instrument at 25 °C by Biotem (Apprieu, France) and Precision Antibody (Columbia, MD, USA). AC‐2 antibody was captured on an anti‐mouse Fc antibody while humanized variants were captured an anti‐human Fc antibody covalently coupled on CM5 sensor chip in an immobilization buffer (10 mm Na‐acetate pH 5.0). This step was followed by binding of Ag (*E. coli* rBAG3, Abcam, Cambridge, UK) at variable concentrations.

### ELISA test for anti‐BAG3 antibodies

96‐well microplates (Thermo Scientific™ MaxiSorp™, cat. no. 442404, Waltham, MA, USA) were coated with 100 μL of solutions containing human recombinant BAG3 protein (1 μg·mL^−1^ in PBS1X) or with specific BAG3 peptides and incubated overnight at 4 °C. The day after, wells were washed with PBS 1X‐0.05% Tween and the blocking of nonspecific sites was performed for 1 h at room temperature in PBS 1X containing 0.5% fish gelatin (Sigma‐Aldrich, Saint Louis, MO, USA). Hence, plates were washed five times with the washing buffer and loaded with hybridoma's supernatants, murine anti‐BAG3 clone AC‐2, humanized mAbs, or mouse sera. Plates were then extensively washed and incubated 30 minutes at room temperature with HRP‐conjugated anti‐mouse IgGs 1 : 2000 (115‐035‐205, Jackson ImmunoResearch, Cambridgeshire, UK) or anti‐human IgG 1 : 20 000 (A0170, Sigma‐Aldrich). Subsequently, TMB solution 1X (eBioscience, San Diego, CA, USA) was added to the wells for the analyte detection. The chromogenic reaction was blocked by acidification with 0.5 m H_2_SO_4_, and the optical density (O.D.) was measured at 450 nm.

### Chemicals, reagents, and kits

FluoroTag™ FITC conjugation kit (FITC1‐1KT) was purchased from Sigma‐Aldrich. Human IL‐6 ELISA (88‐7066‐88) kits were provided by eBioscience.

### Cloning and expression of recombinant BAG3

Human *bag3* CDS (Accession Number NM_004281.3) and murine *bag3* CDS (Accession Number NM_013863.5) were chemically synthesized (GenScript, Leiden, the Netherlands) after gene analysis and optimization for expression in *E. coli* with optimumgenetm software (GenScript). The synthetic DNA fragments, adapted at 5′ and 3′ ends, were cloned into the pAViTag‐N N‐His SUMO Kan Vector (Lucigen, #49044‐1, Middleton, WI, USA) and used to transform *E. coli* Biotin XCell F' cells (Lucigen, #0704‐1). The expression and production of the proteins were then induced and optimized according to the manufacturer instructions. As expected, the recombinant proteins carried a fused N‐terminal biotinylated tag that allowed its capture on streptavidin agarose resin (Thermo Scientific, #20359). The subsequent on‐column cleavage with SUMO Express Protease (Lucigen, #30801‐2) released the full‐length polypeptides that were then further purified on NTA‐Ni resin (Sigma, # P6611) to remove the His‐tagged protease. Pierce High‐Capacity Endotoxin Removal Spin Column (Pierce, #88274, Waltham, MA, USA) was used to obtain endotoxin‐free preparations. Endotoxin concentration was measured by QCL‐1000™ Assay (LONZA; #50‐647U) following the manufacturer instructions.

### Animal studies

The research protocol was approved by the ethics committee in accordance with the institutional guidelines of the Italian Ministry of Health, protocol n. 590/2016‐PR. A total of 20 female CD‐1 nu/nu mice (6 weeks old; Harlan Laboratories, Italy) were used in this experiment and maintained in a barrier facility on HEPA‐filtered racks. 10^6^ MIA PaCa‐2 cells resuspended in 100 μL of a solution of PBS 1X and Matrigel 2 : 1 (Corning, Corning, NY, USA) were injected in the right flank of mice. Once tumor volume average reached the size of 100 mm^3^, animals were randomized into three groups. The experimental groups received 20 mg·kg^−1^ of the BAG3‐H2L4 humanized variants every 48 h. The control group received the same volume of vehicle (PBS 1X) at the indicated times, while the gemcitabine group received the drug 5 mg·kg^−1^ twice a week. Tumor volume was monitored twice a week by a caliper and calculated using the following formula: tumor volume (mm^3^) = (length * width^2^)/2. At the end of the experiment, animals were sacrificed by cervical dislocation by an expert and qualified persons, according to European Federation for Laboratory Animal Science Associations (FELASA). To determine BAG3‐H2L4 half‐life in mouse blood, nude mice bearing MIA PaCa‐2 tumor xenografts were injected intravenously with a single dose of PBS (as vehicle) or BAG3‐H2L4 (20 mg·kg^−1^) and serum samples collected at different time points (1 h, 24 h, 72 h, 7 days, 10 days). BAG3‐H2L4 concentration in serum was measured by ELISA using as capture antigen the human recombinant BAG3 protein and anti‐human IgG‐HRP for detection (Sigma).

### Immunofluorescence

For the evaluation of α‐SMA expression in tumor tissues, samples were paraffin‐embedded and subjected to standard procedures. The immunofluorescence analysis was performed using a mouse monoclonal anti‐α‐SMA antibody from SIGMA (#A2547) and an anti‐mouse Dy‐light 488 (Jackson Laboratories). Nuclei were stained using 4′,6‐diamidino‐2‐phenylindole dihydrochloride (DAPI) (Invitrogen, Carlsbad, CA, USA). Images were acquired in sequential scan mode using the same acquisition parameters when comparing BAG3‐H2L4‐treated (*N* = 2) and control specimens (*N* = 2). Not less than 3 fields per tumor were analyzed using imagej software (Bethesda, MD, USA). Results are shown as % of α‐SMA fluorescent area with respect to the total picture area. BAG3‐H2L4 accumulation in tumor and tissues was evaluated in tissues coming from MIA PaCa‐2 tumor xenografts. Fresh tumors and organ tissues were frozen in a cryo‐embedding medium (OCT, Bio Optica, Milano, Italia), and cryostat sections were incubated with the following antibodies: rat monoclonal anti‐CD31 (550274, BD Pharmingen, San Jose, CA, USA) mixed with rat monoclonal anti‐CD105 (550546, BD Pharmingen), followed by secondary antibody conjugated with Alexa 546 (Invitrogen, Life Technologies, Carlsbad, CA, USA) and Alexa Fluor‐488 conjugated anti‐human IgG (Invitrogen, Life Technologies). Nuclei were stained with DRAQ5 (Alexis, Life Technologies). The representative pictures were elaborated and assembled using Adobe Photoshop 7 and Adobe Illustrator 10.

### Epitope mapping by CLIPS technology

AC‐2 antibody was tested at a concentration of 30 ng·mL^−1^ in a buffer containing 10% of a mix of horse serum and ovalbumin. Preconditioning was performed with a buffer containing 50% of a mix of horse serum and ovalbumin. On the array, SET1 and SET2 are linear peptides of length 15 with an overlap of 14 that cover peptides of BAG3 protein wt sequence or peptides with 2 Ala replacements on positions 12 and 13 with respect to wt sequence. Other four sets of looped peptides of different lengths were designed by using CLIPS technology and employed for this assay.

### Antibody humanization

Humanized AC‐2 variants were obtained as previously described (Sala *et al*., 2013). Briefly, complementarity‐determining regions (CDRs) were identified and grafted onto human antibody framework. The IgG1 isotype was used for all humanized variants. The HC and LC human frameworks are based on the human IgG1 HC G1m17 and human kappa LC Km3. Sixteen humanized antibody variants were constructed by replacing selected residues in the human framework with their AC‐2 counterparts. Recombinant genes were placed into a proprietary transfection‐quality expression vector (EVITRIA AG, Switzerland) and transfected into Chinese hamster ovary (CHO) cells. For small‐/medium‐scale production of antibody variants, transiently transfected CHO was grown and antibody‐containing supernatants were immune‐selected by Protein A FPLC columns by EVITRA AG. Sequences of the humanized anti‐BAG3 variants are described in the patent humanized anti‐BAG3 antibodies (WO2017076878A1).

### Cardiosafety

For cardiosafety assessment of the humanized BAG3‐H2L4 antibody, a total of athymic nude‐Foxn1 mice, 6 months old, were used in this experiment and maintained in a barrier facility on HEPA‐filtered racks. Mice were anesthetized using tiletamine/zolazepam (50/50, 50 mg·kg^−1^); this anesthetic regimen determines a sedation that allows to maintain a physiological heart rate. Then, mice were randomized into three groups: experimental group received 20 mg·kg^−1^ per 100 μL of the monoclonal BAG3‐H2L4 antibody, positive control groups received 2.17 mg·kg^−1^ per 100 μL of doxorubicin (Pfizer, New York, NY, USA), while negative control group received the same volume of vehicle (PBS 1X solution). Mice were treated daily for 7 days. Ejection fraction (EF), shortening fraction (SF), and strain percentage were measured by Vevo 2100 Visualsonics.

### Statistical analysis

Results are expressed as means ± SD or ±SEM Data were analyzed by Student's *t*‐test using medcalc statistical software version 13.3.3 (Ostend, Belgium).

## Results

### Identification of BAG3‐blocking antibodies

**Figure 1:**
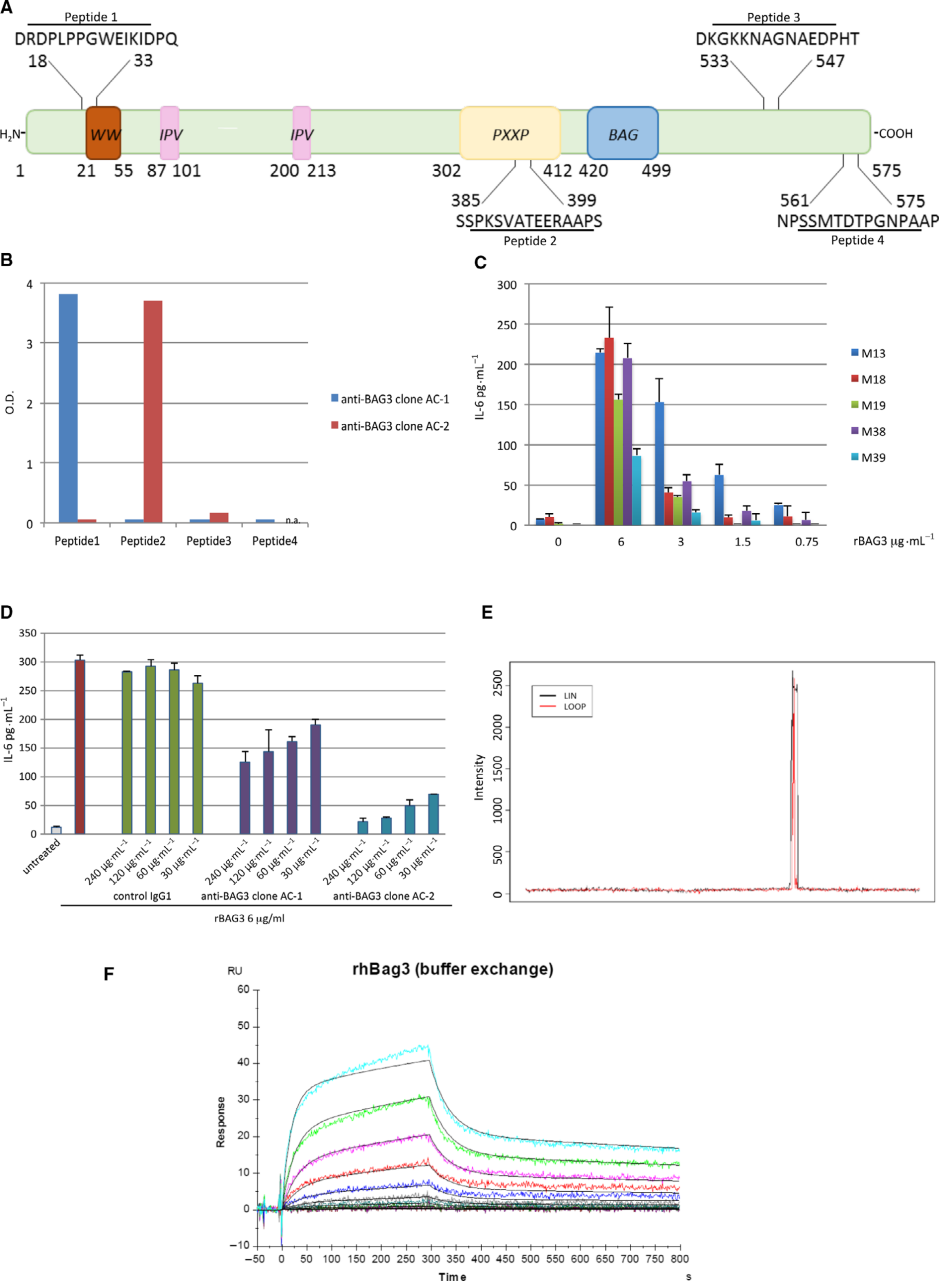
Generation and selection of monoclonal anti‐BAG3 antibodies blocking extracellular BAG3 activity on monocytes/macrophages. (A) Schematic representation of BAG3 with sequences of the peptides used for mouse immunization. (B) ELISA test evaluating binding to BAG3 peptides specified in panel A of antibodies contained in the supernatants from hybridoma clones AC‐1 and AC‐2. (C) IL‐6 production by isolated human monocytes (>98% CD14^+^) from 5 different healthy donors treated with rBAG3 at indicated concentrations for 16 h. Data represent means ± SD of triplicate samples. (D) IL‐6 production by isolated human monocytes (>98% CD14^+^) stimulated with rBAG3 in the presence of increasing concentrations of control IgG1, and anti‐BAG3 clone AC‐1 and AC‐2. Data represent means ± SD of triplicate samples. (E) The graph depicts intensity of signals obtained along the array and shows high binding of the anti‐BAG3 clone AC‐2 in the region surrounding motif PKSVATE for linear (LIN) and looped (LOOP) peptides (black and red traces, respectively). (F) Surface plasmon resonance analysis of mAb AC‐2‐rBAG3 interaction. SPR responses for biosensor chip bound to mAb AC‐2 were recorded for antigen (rBAG3) concentrations ranging from 0.39 to 200 nm. The association (*k*_a_) and dissociation (*k*_d_) constants and affinity (*K*_D_) were calculated using a 2‐state reaction model.

In order to develop specific antibodies blocking BAG3 functions, four spatially distinct BAG3‐derived peptides were chosen to cover different protein domains (Fig. 1A) and used to generate multiple antigenic peptides (MAP). The four selected peptide sequences are specific for BAG3 protein and do not match with other protein sequences, including other members of the BAG protein family (NCBI Reference Sequence: NP_004272.2). BAG3‐MAPs were used for mouse immunization, and nine mother hybridomas were obtained. These were subsequently subcloned in order to obtain single clones specific for each of the four peptides. Two purified hybridoma clones were obtained producing specific mAbs for peptides 1 and 2, while any clone producing mAbs against peptides 3 and 4 was isolated. The antibodies produced by the hybridomas recognizing peptides 1 and 2 were, respectively, named AC‐1 and AC‐2 (Fig. 1B). Then, antibodies were tested for their ability to block BAG3‐dependent monocytes/macrophage activation evaluated as IL‐6 production upon stimulation with a concentration of 6 μg·mL^−1^ of recombinant BAG3 (rBAG3), which was shown to have the highest activating ability (Fig 1C). AC‐2 showed a much higher blocking activity and was selected as a good candidate for a further development (Fig. 1D). Epitope mapping was performed by Pepscan (Lelystad—the Netherlands) and showed that the minimal sequence PKSVATE within peptide 2 was sufficient for the maximal antibody binding (Fig. 1E). Finally, we used surface plasmon resonance (SPR) analysis to determine the KD value of the antibody–antigen interaction (14.4 ± 1.3 × 10^−9^ m) (Fig. 1F).

### Humanized antibodies screening and lead selection

**Figure 2:**
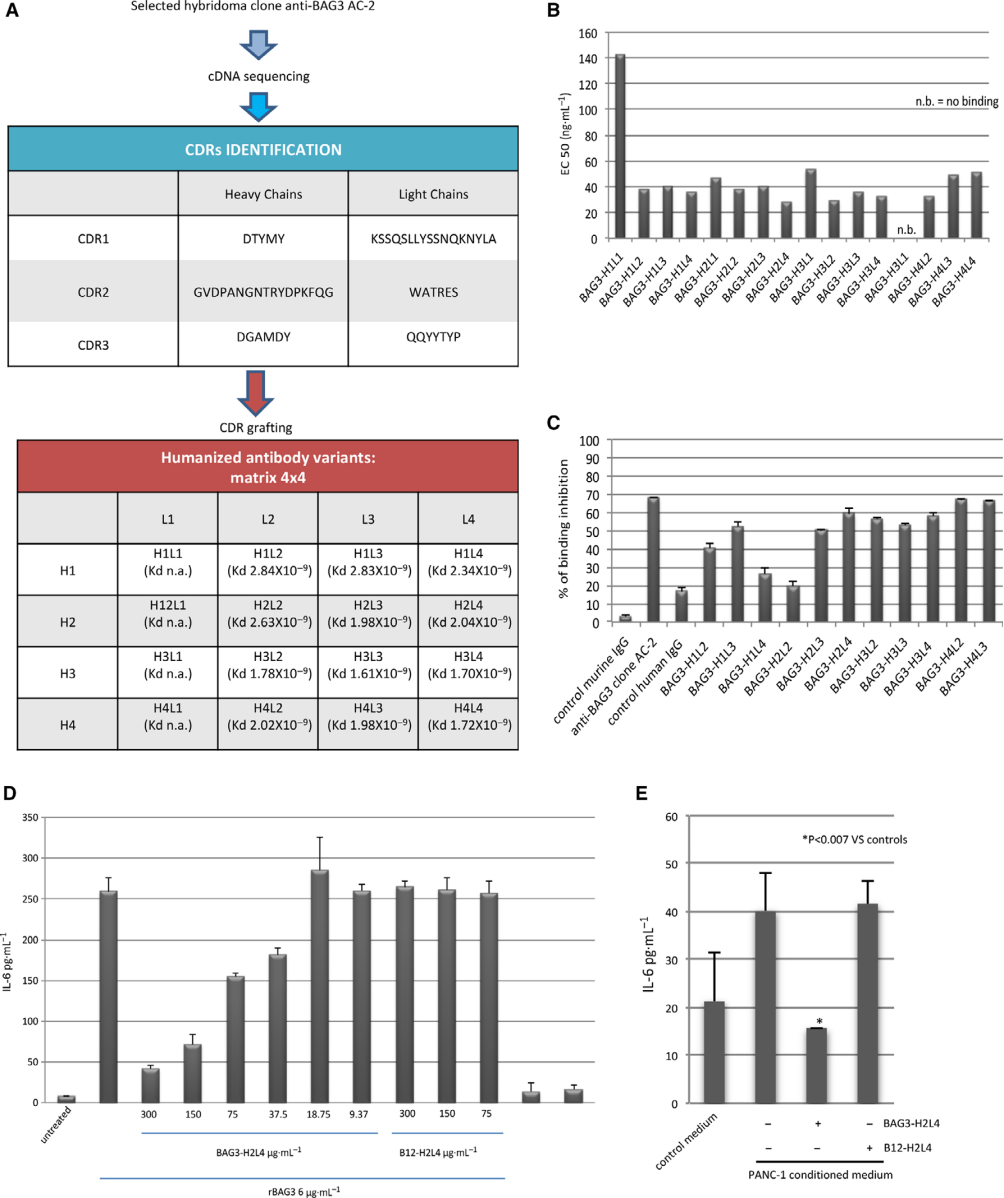
Anti‐BAG3 humanized antibody lead selection. (A) Schematic representation of the steps of the humanization process. Sequences of CDRs in heavy and light chain of the antibody are reported as well as the KD values for rBAG3 binding for each variant. (B) Screening for rBAG3 binding ability of humanized antibody variants by direct ELISA test. Histograms represent EC50 values obtained using scalar dilutions of the antibodies (500 ng·mL^−1^ to 15.6 ng·mL^−1^). (C) Inhibition of FITC‐rBAG3 protein binding to J774 A.1 cells by the different humanized antibody variants. Histograms represent % of binding inhibition evaluated by flow cytometry as mean fluorescence intensity changes. Data represent means ± SD of triplicate samples. (D) IL‐6 production by isolated human monocytes (>98% CD14+) stimulated with rBAG3 in the presence of increasing concentrations of BAG3‐H2L4 antibody. As a control, an unrelated recombinant human IgG1 carrying the same heavy and light chains of BAG3‐H2L4 antibody was used (B12‐H2L4). Data represent means ± SD of triplicate samples. (E) Isolated human monocytes (>98% CD14+) were stimulated using conditioned medium of PANC‐1 subconfluent cultures for 16 h alone or in the presence of BAG3‐H2L4 or control B12‐H2L4 (200 μg·mL^−1^). *P* was calculated by Student's *t*‐test.

AC‐2‐derived humanized antibodies were generated by complementarity‐determining region (CDR) grafting as described in the Methods section. CDR sequences used are shown in Fig. 1A. Four different heavy (H) and light (L) chain variants were combined in a four‐by‐four matrix to generate 16 different antibodies (H from 1 to 4/L from 1 to 4) (Fig. 2A). To identify the antibody variants with the highest activity, we carried out an initial screening evaluating: (a) KD by SPR analysis (Fig. 2A); (b) binding to full‐length BAG3 by direct ELISA test (Fig. 2B); (c) inhibition of BAG3‐FITC binding to cell surface of macrophages of the murine cell line J774A.1 (Fig. 2C). All antibodies containing the L1 chain were excluded since they showed a low binding affinity. Among the remaining variants, BAG3‐H2L4 and BAG3‐H4L2 showed the best results in all three tests. However, since it was possible to obtain higher yields for BAG3‐H2L4 production, we chose to further develop this variant. BAG3‐H2L4 was capable of blocking BAG‐3‐dependent IL‐6 release by human monocytes in a dose‐dependent manner (Fig. 2D) as well as monocyte activation when cultured using PDAC cells (PANC‐1) conditioned medium (Fig. 2E).

### BAG3‐H2L4 therapeutic activity

**Figure 3:**
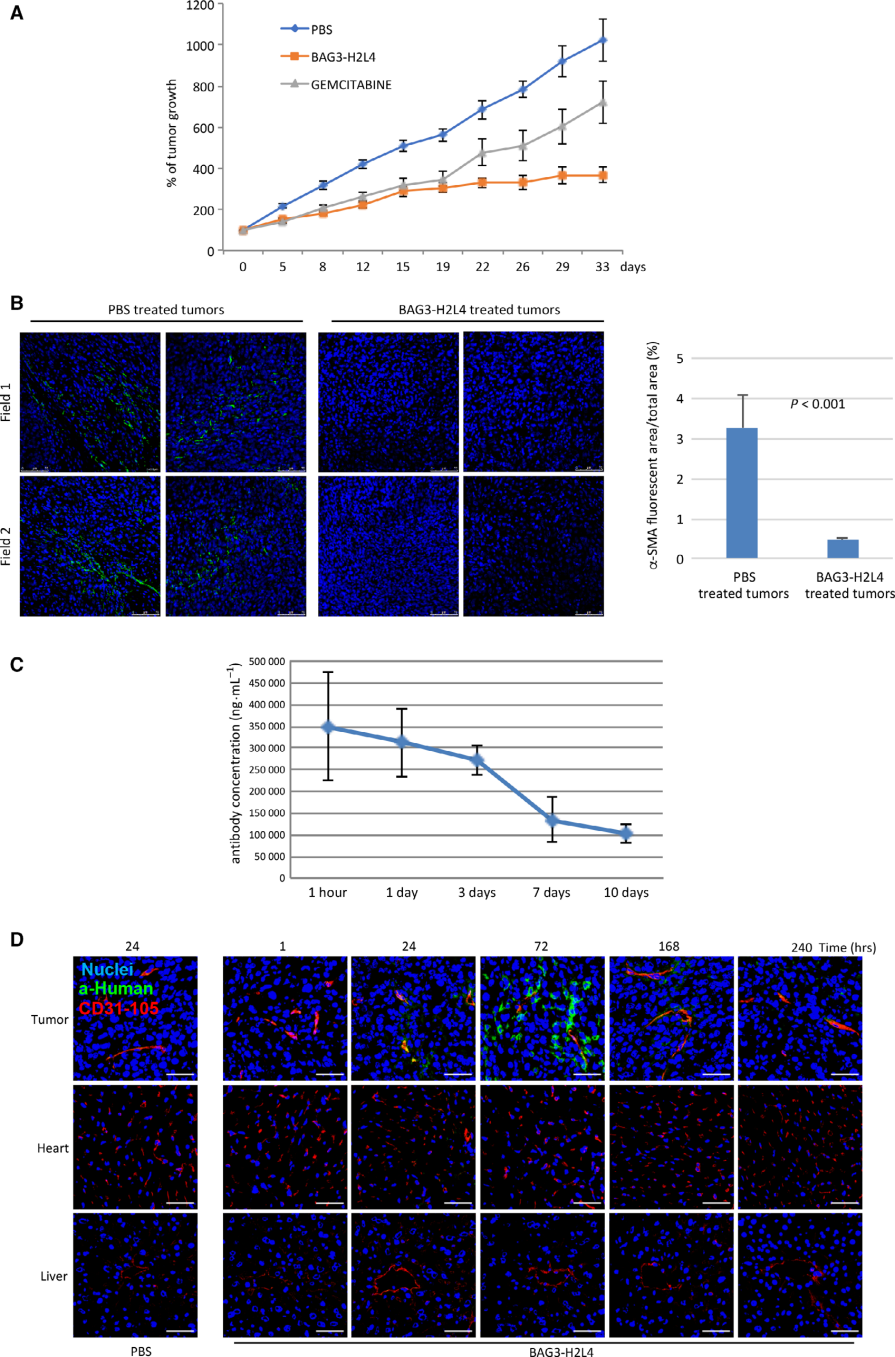
BAG3‐H2L4 therapeutic activity. (A) Exponentially growing MIA PaCa‐2 cells were injected into the right flank of the recipient mice. Animals were treated twice a week with 20 mg·kg^−1^ BAG3‐H2L4 or gemcitabine (5 mg·kg^−1^) or with vehicle alone (PBS). Tumor growth was assessed, as described in the Materials and Methods section. Results are expressed as percentage fold change (±SEM). (B) Tumor specimens (PBS‐treated tumors *N* = 2; BAG3‐H2L4‐treated tumors *N* = 2) were analyzed by immunofluorescence using anti‐α‐SMA antibody. Nuclei were counterstained with DAPI (40× magnification‐scale bars 75 μm). Results were quantified as mean percentages of α‐SMA fluorescent area with respect to the total picture area using imagej software. Error bars indicate SD *P* was calculated by Student's *t*‐test. (C) BAG3‐H2L4 concentration in mouse sera was analyzed by ELISA. The graph depicts means sera concentrations from 3 different animals for each time point (±SD). (D) Representative images from tumor, heart, and liver sections collected from mice treated with BAG3‐H2L4 or the vehicle (as control). Slides were stained with anti‐CD31‐105 and anti‐human IgGs (scale bars 50 μm).

In order to analyze the therapeutic activity of the humanized BAG3‐H2L4 antibody, we grafted the human PDAC cell line, MIA PaCa‐2, in immune‐deficient mice and, once tumors were established, treated mice with 20 mg·kg^−1^ of BAG3‐H2L4 or 5 mg·kg^−1^ of gemcitabine, or PBS. While a modest response was observed in mice treated with gemcitabine, a significant (*P* < 0.05) tumor growth inhibition was detected in BAG3‐H2L4‐treated mice (Fig. 3A). Of note, no significant weight loss was observed during the antibody treatment (data not shown).

As previously shown using the murine anti‐BAG3 AC‐2 antibody (Iorio *et al*., 2019), also BAG3‐H2L4 treatment affects the tumor microenvironment resulting in a reduction of α‐SMA‐positive fibroblasts (Fig. 3B), effectively recapitulating the functional effects of the original murine version *in vitro* and *in vivo*.

### PK, tissue distribution, and preliminary toxicology studies of BAG3‐H2L4

The PK profile of BAG3‐H2L4 antibody was evaluated in nude mice harboring PDAC tumors generated as described above. Following i.v. injection, BAG3‐H2L4 *t*_1/2_ and AUC values were found to be 127 77 h and 66 848 (mg*h·mL^−1^), respectively (Fig. 3C). Moreover, we observed a time‐dependent antibody accumulation in tumors, indicating target cell accessibility to BAG3‐H2L4 *in vivo* (Fig. 3D).

As expected, BAG3‐H2L4 recognizes the murine form of BAG3 (see Fig. S1) similarly to the murine anti‐BAG3 antibody AC‐2 (Rosati *et al*.,^2015^). More importantly, we show that it does not accumulate in healthy organs known to express intracellular BAG3 (Homma *et al*., 2006) such as heart and liver (Fig. 3D).

Moreover, to further exclude cardiotoxicity associated with anti‐BAG3 treatment, we treated athymic nude‐Foxn1 nu/nu mice with BAG3‐H2L4, doxorubicin (positive control) or vehicle (negative control) daily for 7 days and measured the ejection fraction (EF), shortening fraction (SF), and strain percentage. As shown in Fig. S1, doxorubicin treatment resulted in reduction of all the heart functionality parameters measured, as expected, while treatment with BAG3‐H2L4 had no effect, thus suggesting a lack of cardiotoxicity of the anti‐BAG3 therapy.

## Discussion

BAG3 protein is emerging for its role as a potential target for cancer therapy (Guerriero *et al*., 2017; De Marco *et al*., 2018; Esposito *et al*., 2017; Chiappetta *et al*., 2014; Guerriero *et al*., 2014; Rosati *et al*., 2012a,b; Franco *et al*., 2012; Festa *et al*., 2011; Chiappetta *et al*., 2012; Shi *et al*., 2018; Nymoen *et al.,* 2015; Colvin *et al*., 2014; Li *et al*., 2015; Shi *et al*., 2018; Yunoki *et al*., 2017; Bartsch *et al*., 2016; Yeo *et al*., 2015; Xiao *et al*., 2014; Aust *et al*., 2013) not only for its intracellular functions, but also for its role as a secreted protein signaling from tumor to its microenvironment. We have previously shown, in several murine models, that blocking BAG3 function results in lowering macrophage infiltrate and cyto/chemokine load in PDAC, thus reducing tumor growth and metastatic spreading (Rosati *et al*., 2015). Furthermore, blocking BAG3 activity increases immune check point inhibitor‐based therapies' efficacy (Iorio *et al*., 2018). Moreover, a different approach has been proposed to inhibit BAG3 activity for cancer therapy using a small molecule. In fact recently, the first selective BAG domain modulator of BAG3 has been proposed as a novel candidate for the development of a new class of chemotherapeutic agents (Terracciano *et al*., 2018); this 2,4‐thiazolidinedione derivative, by interfering with the binding between BAG3 and Hsp70, reduces cancer cell proliferation.

Mice antibodies cannot be used for therapy in humans due to human antimurine response; therefore, humanization process is the first step required for the development of a potential drug candidate.

Here, we describe the characterization of a humanized anti‐BAG3 antibody generated through recombinant DNA technology. Sixteen humanized variants of the murine AC‐2 antibody were generated by CDR grafting. The antibody variant named BAG3‐H2L4 was selected as the lead compound following screening based on affinity to the target and blocking activity. BAG3‐H2L4 demonstrated significant therapeutic activity in a PDAC xenograft model interfering with the interaction between cancer and its microenvironment. Of note, the antibody accumulated in a specific manner in the tumor but not in normal tissues (Rosati *et al*., 2015). These results suggest that secreted BAG3 is mainly present in the tumor microenvironment and not in tissues, such as the heart, normally expressing significant levels of the intracellular protein. Indeed, no cardiotoxicity was observed after administration of the antibody in mice. The observed absence of cardiotoxicity might represent an advantage in comparison with other anti‐BAG3‐blocking agents based on small molecules that might also interfere with its intracellular functions.

## Conclusion

Pancreatic ductal adenocarcinoma incidence is increasing in industrialized countries. Despite the efforts made in recent decades, PDAC remains a type of incurable cancer whose 5‐year average survival does not exceed 7.1% (Siegel *et al*., 2015). Therefore, innovative and effective therapies represent an urgent medical need. Here, we describe an anti‐BAG3 humanized mAb able to block BAG3 activity and PDAC tumor growth, providing evidence that BAG3‐ H2L4 humanized antibody is a potential candidate for BAG3‐based targeted therapy in the clinical setting.

## Conflict of interest

AB, MDM, MF, AF, LM, AR, VDL and MCT are shareholders of BIOUNIVERSA s.r.l. that own anti‐BAG3 antibodies. The other authors have no competing interests.

## Author contributions

AB, MDM, MF, AF, VI, LG, DE, DR, AL, PB, CA, VD, and AR conducted experiments and statistical analysis. LM, GS, AR, VDL, and MCT designed the experiments. GS and AR wrote the paper with input from all authors; and VDL and LM supervised the project.

## Supporting information


**Fig. S1.** (A) Murine rBAG3 was loaded at quantities indicated on a SDS/PAGE and then proteins transferred to a nitrocellulose paper. BAG3‐H2L4 was used for the immunoblot at a concentration of 20 μg·mL^−1^. (B) BAG3‐H2L4 or the unrelated B12‐H2L4 antibodies were used to immunoprecipitate the murine rBAG3. Subsequent immunoblot was performed with a rabbit polyclonal anti‐BAG3 raised against the full length human rBAG3. (C) Ejection fraction (EF), shortening fraction (SF), and strain percentage (SP) from control, doxorubicin or BAG3‐H2L4 treated mice are expressed as means (±SD). (T0 = before treatment; T1 = 3 days treatment; T2 = 7 days treatment). Significant differences in measurements of the three different treatment groups were assessed by using student's *t* test. *P and **P are referred to doxorubicin treatment compared to BAG3‐H2L4 treatment at T1 and T2, respectively.
