# Supplementary figures and images for: Development of an anti‐BAG3 humanized antibody for treatment of pancreatic cancer
Source: Mol Oncol. 2019 May 17;13(6):1388–99. doi: 10.1002/1878-0261.12492 (PMC6547619; doi:10.1002/1878-0261.12492)

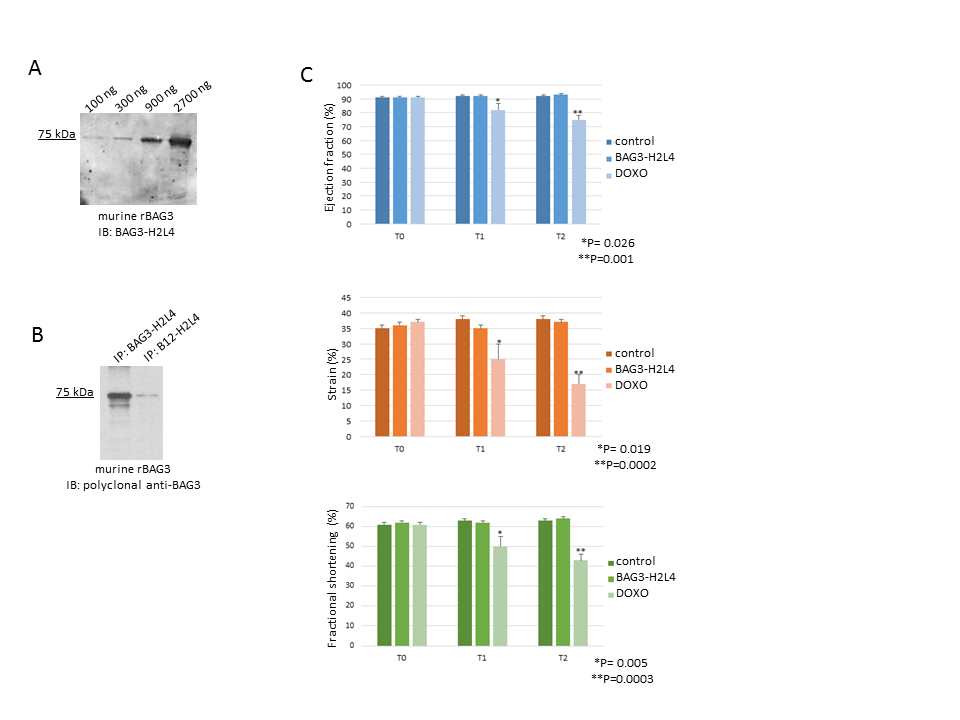

Supplement: Supplementary file 1 — Fig. S1. (A) Murine rBAG3 was loaded at quantities indicated on a SDS/PAGE and then proteins transferred to a nitrocellulose paper. BAG3‐H2L4 was used for the immunoblot at a concentration of 20 μg·mL−1. (B) BAG3‐H2L4 or the unrelated B12‐H2L4 antibodies were used to immunoprecipitate the murine rBAG3. Subsequent immunoblot was performed with a rabbit polyclonal anti‐BAG3 raised against the full length human rBAG3. (C) Ejection fraction (EF), shortening fraction (SF), and strain percentage (SP) from control, doxorubicin or BAG3‐H2L4 treated mice are expressed as means (±SD). (T0 = before treatment; T1 = 3 days treatment; T2 = 7 days treatment). Significant differences in measurements of the three different treatment groups were assessed by using student's t test. *P and **P are referred to doxorubicin treatment compared to BAG3‐H2L4 treatment at T1 and T2, respectively. [file MOL2-13-1388-s001.tif]
